# Control Capabilities of Myoelectric Robotic Prostheses by Hand Amputees: A Scientific Research and Market Overview

**DOI:** 10.3389/fnsys.2015.00162

**Published:** 2015-11-30

**Authors:** Manfredo Atzori, Henning Müller

**Affiliations:** Information Systems Institute, University of Applied Sciences Western Switzerland (HES-SO Valais)Sierre, Switzerland

**Keywords:** electromyography, prosthetics, rehabilitation robotics, machine learning

## Abstract

Hand amputation can dramatically affect the capabilities of a person. Cortical reorganization occurs in the brain, but the motor and somatosensorial cortex can interact with the remnant muscles of the missing hand even many years after the amputation, leading to the possibility to restore the capabilities of hand amputees through myoelectric prostheses. Myoelectric hand prostheses with many degrees of freedom are commercially available and recent advances in rehabilitation robotics suggest that their natural control can be performed in real life. The first commercial products exploiting pattern recognition to recognize the movements have recently been released, however the most common control systems are still usually unnatural and must be learned through long training. Dexterous and naturally controlled robotic prostheses can become reality in the everyday life of amputees but the path still requires many steps. This mini-review aims to improve the situation by giving an overview of the advancements in the commercial and scientific domains in order to outline the current and future chances in this field and to foster the integration between market and scientific research.

## Introduction

It is estimated that 41,000 persons were living with a major loss of an upper limb in 2005 (Ziegler-Graham et al., 2008). A hand amputation is one of the most impairing injuries and it can dramatically affect the capabilities of a person. Recent scientific and commercial advances in man-machine interfaces are promising and suggest that dexterous, naturally controlled, proportional and simultaneous robotic prostheses could be reality in the future of amputees. Nevertheless, the outline of the situation in the market and scientific field is complex and the path to naturally controlled prostheses still requires several steps.

Man-machine interfaces have been developed to control hand prostheses via the brain (Lebedev and Nicolelis, 2006), peripheral nerves (Navarro et al., 2005) or the muscles (Cipriani et al., 2011). The first two methods are promising but they usually require invasive procedures to obtain robust performance, thus they are currently applied only in scientific research. The third method (surface electromyography, sEMG) is probably the most widely used both in commercial settings and in scientific research.

Myoelectric hand prostheses with many degrees of freedom and very good mechanical capabilities are now commercially available. However, prosthetics companies target most of their communication efforts to end users. Thus they highlight the practical capabilities of the hands, but they usually do not provide information regarding the technical functionalities and specifications of the prostheses that can be exploitable by academic researchers. Previous papers presented some hand prostheses in detail (Belter et al., 2013) but the market changes quickly.

The scientific research field is even more complex and quickly changing. Many papers have been written in scientific research about the natural control of robotic hands by intact and transradial hand amputated subjects. Most of the methods rely on the use of sEMG and of pattern recognition or proportional control algorithms. The first commercial products exploiting pattern recognition to recognize the movements have recently been released. Targeted muscle reinnervation (TMR) can allow the exploitation of these methods even on subjects with above-elbow amputations. Benchmark databases to compare the performance of different methods and setups have been released (Atzori et al., 2014a). However, several steps are still required to obtain proportional, naturally controlled, robust and usable robotic hand prostheses (bionic hands).

Since the market and the scientific field are so complex and changing so quickly, it can be difficult to have a complete overview of them and to remain constantly updated in both fields. This mini-review aims to be a resource for young and experienced researchers in academia and prosthetic companies by providing a synthetic but complete overview of the current level of advancement in the commercial and scientific reality.

## Market Outline

A relatively wide choice of devices is available to restore the capabilities of hand amputees by myoelectric robotic prostheses. Such devices are continuously evolving according to technology, scientific research, market needs and user requirements. The devices usually include two main parts: prosthetic hands and control systems.

### Prosthetic Hands

Currently, hand prostheses include cosmetic prostheses, kinematic prostheses and myoelectric prostheses. Cosmetic prostheses offer esthetical and psychological support. Kinematic prostheses also have functional capabilities, since the user can control the opening and closing of a gripper hand through the motion of the shoulder. Myoelectric prosthesis users can control a battery-powered hand through the electrical signal emitted by the remnant muscles, usually located in the forearm.

The continuous improvements in the field and the different targets and aims of the papers published by the companies can make it difficult for researchers to remain updated with the capabilities of available prostheses. For example, Belter et al. (2013) performed a very thorough description of the mechanical properties of prosthetic hands produced by four companies, but in less than 2 years several companies produced new versions or made substantial changes to the products from a mechanical or electronic point of view. Thus, the market and research achievements often remain disconnected.

Many prosthetic hands are commercially available. However, few have the capability to reproduce many movements. The following selection represents some of the currently most advanced hand prostheses and gives a representation of different companies and approaches: (1) Touch Bionics i-limb Quantum; (2) Otto Bock Michelangelo; (3) Steeper Bebionic v3; and (4) Vincent hand Evolution 2. Table 1 summarizes the most important features that can be useful in a laboratory. The features are grouped into the following four categories: general technical data, dexterity related features, force related features and control related features.

**Table 1:** **Characteristics of the examined prosthetic hands**.

	Company name Prosthesis model	Touch Bionics i-limb Quantum	Otto Bock Michelangelo with Axon Bus Technology	Steeper Bebionic v3	Vincent GmbH Evolution 2
General technical data	Weight (without battery)	474–515 g	~510 g	550–598 g (365–390 g small hand)	380–410g
	Operating voltage	7.4 V	11.1 V	7.4 V	6–8 V
	Battery type	Lithium polymer	Li-Ion	Li-Ion	Li-Pol
	Battery capacity	1300–2400 mAh	1500 mAh	1300–2200 mAh	1300–2600 mAh
	Number of actuators	6	2	5	6
Dexterity	Active fingers	5 independent	3	5 independent	5 (+12 a*ctive joints*)
	Thumb rotation	Powered	Powered	Manual	Powered
	Total number of grip patterns	24	7	14	20
	Grip patterns available at any moment	7	7	11	20
	Flexible wrist	Available	Included	Available	Available
	Rotating wrist	Available	Available	Available	Available
	Rotating wrist	*(active or passive)*	*(active or passive)*	*(active or passive)*	*(only passive)*
	Full closing time	0.8 s (0.7 s small hand)	0.37 s	0.5–1 s	0.8 s
	Finger position encoders	No	2 *motor position encoders*	5 *(one in each actuator)*	2 *(in thumb actuators)*
Force	Power grip	100–136 N	~70 N	140.1 N (280 N small hand)	60 N
	Lateral pinch	40 N (60 N small hand)	~60 N	26.5 N (53 N small hand)	15 N
	Adaptive Grip	Yes	Yes	Yes	Yes
	Falling object prevention	Active *(auto-grasp, based on accidental sEMG signal detection)*	No	Active *(auto-grip, based on finger position encoders)*	Passive*(spring load)*
	Proportional control	Yes	Yes	Yes	Yes
	N° of electrodes	1–2	1–2–3	1–2	1–2 wired
Control	Movement control type	Movement triggers, mobile app, bluetooth grip chips, favorite environment, gesture control	Sequential, 4-channel control	Sequential, Morph RFId GRIP selection compatible	Single trigger or Vincent Morse code
	Movement command	Hold open, double impulse, triple impulse, co-contraction	Different switching modes available, fast and high signal controls rotation in 4-channel control	Co-contraction/open-open signal	Hold signal (opening or closing), double signal, co-contraction, alternating signal
	Particular features	Various control methods thumb rotating manually and automatically	Sensor hand speed *(stiff and harder finger tips)*; Fragile objects grasping	Fully free flexing fingers	Very low weight
	Feedback	No	No	Audible beeps and/or vibration *(grip changes)*	Vibration *(force detected via motor current and DMS sensors)*

### Control Systems

**Figure 1:**
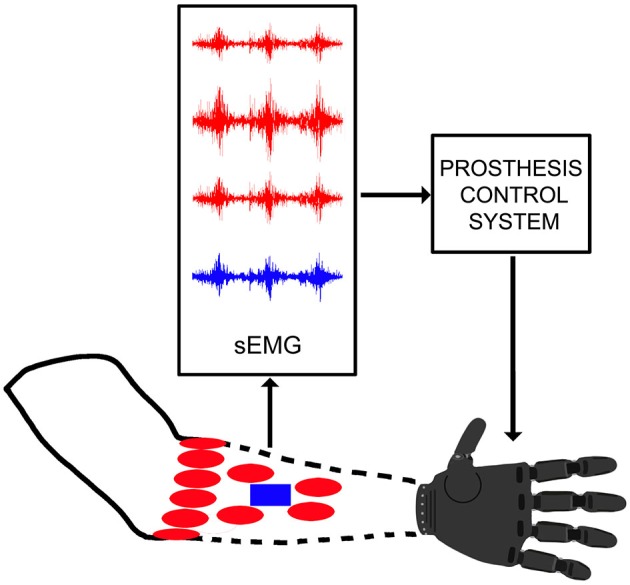
**Scheme of a generic myoelectric control system: (i) for commercial prosthesis without pattern recognition (blue rectangle); and (ii) for research (or control system with pattern recognition; red ellipses).** The same architecture is assumed in the external forearm.

Usually two or three sEMG electrodes are located in the socket in correspondence to specific muscles (Figure 1). A myoelectric impulse (i.e., an increase in the amplitude of the electrical signal emitted by the muscles) is used to open and close the prosthetic hand. The number of movements can be increased employing specific (e.g., sequential) control strategies. Such control strategies are usually still far from being natural, thus controlling prostheses requires a high level of skill and a training procedure. Control problems contribute to the scarce capabilities and acceptance of sEMG prostheses (Atkins et al., 1996), but they are likely promising for improvements in a near future.

In Table 1 we summarize some of the most important control related features for the considered prosthetic hands including: number of electrodes, movement control type, movement command and particular features of each control system. As can be noticed in Table 1, despite the mechanical characteristics of the prosthesis allowing to reproduce up to 24 hand movements, the control systems rely in most cases on few (1–3) electrodes and on sequential control strategies or on specific movement triggers (in some cases tunable through a mobile app or other strategies). In sequential control strategies, a specific signal (for example, a simultaneous activation of two sEMG electrodes, usually called co-contraction) is used to switch between a set of predefined movements. In movement triggers on the other hand specific patterns of electrode activation are related to specific movements of the prosthesis. The mentioned methods are not natural, in the sense that they do not correspond to the movement that the subject would have thought to do before the amputation. However, they offer robust results, which is one of the main needs in real life.

Several of the considered prostheses include external sources of information as well. In particular, Touch Bionics i-limb Quantum recently introduced gesture control (recorded via gyroscope, accelerometer and magnetometer) and grip chips (that use blue-tooth chips attached to specific objects) to perform movement selection, while Steeper Bebionic exploits finger position encoders to perform falling object prevention. Sometimes research achievements translate to clinical practice too. In 2013 a pattern recognition system similar to the ones described in the scientific literature was made commercially available (http://www.coaptengineering.com/). The Coapt system can include up to eight sEMG electrodes. It is generic and it is typically set up to control the number of powered DOFs the patient’s prosthesis has. That is, if a powered elbow, wrist, and terminal device are built into the prosthesis then the Coapt system is set to control these. If, however the prosthesis only has a powered terminal device and/or wrist, the Coapt system is set up for those DOFs. Wherever possible, Coapt performs natural control. The technician is encouraged to work with the patient to determine which are the most physiological, repeatable, consistent, and intuitive movements to use for control. Slight variations can be attempted if necessary, also through re-calibration procedures. The number of natural grasping patterns that can be achieved varies. According to Coapt, typically users can select between 3–6 naturally. It should be noted that the physical interconnection of the Coapt system and several prostheses has yet to be implemented. An example of movement-triggered control that we received by Coapt is the following one:

1. Hand closing: closing prosthesis.
2. Hand opening: opening prosthesis.
3. Wrist clockwise/counterclockwise rotation: powered wrist clockwise/counterclockwise rotation.
4. Double impulse of natural hand opening: grip A.
5. Triple impulse of natural hand opening: grip B.
6. Holding the hand open: grip C.
7. Single impulse of natural hand opening: grip D.

## Scientific Research Outline

Many papers have been written in scientific research about the control of robotic hands and prostheses by intact and hand amputated subjects.

Usually several electrodes are placed on the forearm of the subject to record the myoelectric signals (Figure 1) with a dense sampling approach (Fukuda et al., 2003; Tenore et al., 2009; Li et al., 2010) or a precise anatomical positioning strategy (De Luca, 1997; Castellini et al., 2009a). The most common control procedures can be subdivided into pattern recognition or proportional control approaches, which can be applied to sEMG and multimodal signals.

Pattern recognition algorithms are used to classify the movement that the subject aims to perform according to a label (Scheme and Englehart, 2011). Pattern recognition results provided in several cases classification accuracy over 90–95% on less than 10 classes (e.g., Castellini et al., 2009b), however average results are usually below 80–90% (Peerdeman et al., 2011). Movement classification methods require movement labeling and they are restricted to a predetermined set of hand movements. Simultaneous pattern recognition has been studied recently (Jiang et al., 2013b; Ortiz-Catalan et al., 2013; Young et al., 2013), however usually such procedures consider simultaneous motions as new classes, thus they can reduce the robustness of the classifier.

Proportional and simultaneous control of a large number of degrees of freedom of the prosthesis can allow achieving more natural and dexterous control using unsupervised or supervised methods (Fougner et al., 2012; Farina et al., 2014). Unsupervised methods are usually based on signal factorization (e.g., through Non-Negative Matrix Factorization, NMF), they require a short calibration phase and they are relatively independent on the number and exact location of the electrodes (Jiang et al., 2009, 2014a,b; Muceli et al., 2014). Supervised methods (Nielsen et al., 2011; Muceli and Farina, 2012; Ameri et al., 2014a,b; Gijsberts et al., 2014b; Hahne et al., 2014) are usually based on regression techniques (e.g., Linear Regression, LR, Artificial Neural Networks, ANN, Support Vector Machines, SVM) that require a reliable ground truth for hand kinematics. This is easy for intact subjects (e.g., using data gloves), but it can be difficult for amputees, for whom the ground truth can be acquired only via bilateral mirrored contractions (Nielsen et al., 2011) or via visual cues (Ameri et al., 2014a,b). Recently, semi-supervised methods (NMF) and supervised methods (LR, ANN) were compared to evaluate the impact of precise kinematics estimation for accurately completing goal-directed tasks (Jiang et al., 2014b). The results showed that, although the three algorithms’ mapping accuracies were significantly different, their online performance was similar. These results underline the hypothesis that good proportional myoelectric control can be achieved by the interaction and adaptation of the user with the myoelectric controller through closed-loop feedback. The same hypothesis is also demonstrated in other recent papers on multiple degrees of freedom for intact subjects (Pistohl et al., 2013; Antuvan et al., 2014) and hand amputees (Jiang et al., 2014a). Despite most of the proportional studies concentrating on full hand movements (e.g., hand supination, pronation, rotation, flexion, extension), proportional and simultaneous control has a strong potential for decoding finger kinematics as well. In particular, recent work described average correlation coefficients of up to 0.9 for the estimation of single finger movements (Smith et al., 2008) and 0.8 for the estimation of simultaneous and complex movements (Ngeo et al., 2014).

Also in scientific research, additional sources of information can be used to improve the performance of myoelectric control. Computer vision has been integrated to predetermine the type and size of the required grasp in relation to the object (Došen et al., 2010; Markovic et al., 2014). Accelerometers showed excellent capabilities to recognize hand movements using pattern recognition and regression methods, both alone and in combination with sEMG electrodes (Atzori et al., 2014b; Gijsberts et al., 2014a; Krasoulis et al., 2015).

A common problem in the field is that often the studies are highly specific and they are not directly comparable, due to different acquisition setups, protocols and analysis pipelines. Moreover, often the datasets are not publicly available. The NinaPro project (Atzori et al., 2015) released a publicly available benchmark with EMG, kinematic and dynamic data sources from intact and amputated subjects to help the scientific community to overcome control problems (http://ninaweb.hevs.ch/). Ninapro was recently used to evaluate regression methods for the continuous decoding of finger movements from sEMG and accelerometry (Krasoulis et al., 2015), to apply Dynamic time warping (DTW) in the context of myoelectric control (AbdelMaseeh et al., 2015) and to present the *Movement Error Rate*, an alternative to the standard window-based accuracy in pattern recognition (Gijsberts et al., 2014a).

Many factors can theoretically influence sEMG controlled prosthesis, including anatomical characteristics of the subjects (Farina et al., 2002), training in using myoelectric prostheses (Cipriani et al., 2011), clinical parameters of the subjects (e.g., level of the amputation, phantom limb sensation intensity; Atzori et al., in press), fatigue, sweating, changes in electrode or arm positioning, surgical procedures used during the amputation and even cortical reorganization. However, few studies addressed these effects.

Implanting intramuscular EMG-recording devices reduces the number of parameters affecting the EMG signal and it can improve simultaneous control of multi-DOF prosthetic wrist and hand (Smith et al., 2014, 2015).

TMR is a surgical procedure that redirects the nerves that used to control the muscles of the hand to innervate accessory muscles from which surface sEMG is recorded. Impressive results have been obtained with this method, especially in persons with above-elbow or shoulder amputations (Kuiken et al., 2009). The same technique has also been applied on muscles transferred to the forearm to better integrate with traditional commercial prostheses (Aszmann et al., 2015).

The opposite neural direction, i.e., transferring information from the hand prosthesis to the brain, has been studied in several papers as well. Several attempts have been performed using non-invasive or invasive methods. Electrocutaneous and vibratory stimulation channels have been extensively studied in the past Szeto and Saunders (1982). TMR represents a promising solution also in this case, since it theoretically allows a certain amount of sensory feedback (Marasco et al., 2009). However, to date, the only example of real-time use of neural interfaces for the effective bidirectional control of dexterous prosthetic hands performing different grasping tasks is given by Raspopovic et al. (2014).

Despite the achievements described in this article, there are still several challenges before amputees can benefit from the mentioned signal processing developments (Jiang et al., 2012). First, robustness is probably the most important and challenging problem, in particular for simultaneous and proportional control. Second, the sensory-motor loop should be closed with proper feedback systems, thus opening new possibilities for effective and intuitive prosthetic control. Third, most of the studies are performed in controlled laboratory conditions with non-amputated subjects, which do not adapt to several different real life conditions of amputees (Fougner et al., 2011; Jiang et al., 2013a; He et al., 2015a,b).

## Conclusions

Hand amputation can dramatically affect the capabilities of a person. The augmentation of the functionalities of the nervous and muscular system through external devices can already improve the situation of amputees. The market and the scientific field are complex and changing quickly, thus it is often difficult for young researchers to have a complete overview of them, as well as for experienced researchers to remain constantly updated in both the fields. In this mini review, we provide a synthetic but complete overview of the current level of advancement in the commercial and scientific reality, addressing each field in a specific section.

The commercial outline highlights the existence of very advanced prosthetic hands and control systems. Four of the most advanced prosthetic hands were analyzed, showing important mechanical and control differences. In particular, the number of actuators ranges between 2 (Otto Bock Michelangelo), 5 (Steeper Bebionic 3) and 6 (Touch Bionics i-limb Quantum and Vincent Evolution 2) while the number of finger position encoders ranges between 0 (Touch Bionics i-limb Quantum), 2 (Otto Bock Michelangelo, Vincent Evolution 2) and 5 (Steeper Bebionic 3). The first commercial control system based on pattern recognition has been released and it seems a great advancement with respect to previous ones. However natural, proportional and simultaneous control of a large number of degrees of freedom is currently not available.

The scientific research outline shows a large variety of control methods and several possible improvements. Pattern recognition, proportional control and TMR are extremely promising. Common sEMG data resources and benchmarks have been proposed recently to compare different sEMG analysis methods. Most of the factors that can theoretically affect the control of myoelectric prostheses, such as clinical data (e.g., level of the amputation, phantom limb sensation intensity) were recently studied. Finally sensorial feedback recently showed very promising advancements.

In conclusion, the path to proportional, naturally controlled, robust and usable robotic hand prostheses with sensorial feedback (bionic hands) seems to be well initiated and extremely promising for the coming years even though it is still a challenging work in progress.

## Conflict of Interest Statement

The authors declare that the research was conducted in the absence of any commercial or financial relationships that could be construed as a potential conflict of interest.
